# Assessment of Beverage Trends and Replacing Nondairy Caloric Beverages with Milk at Meals across Childhood Improves Intake of Key Nutrients at Risk of Inadequate Consumption: An NHANES Modeling Study

**DOI:** 10.1016/j.cdnut.2023.102020

**Published:** 2023-10-18

**Authors:** Kristin Ricklefs-Johnson, Matthew A. Pikosky, Christopher J. Cifelli, Kristin Fulgoni, Victor L. Fulgoni, Sanjiv Agarwal

**Affiliations:** 1 National Dairy Council, Rosemont, IL, United States; 2 Nutrition Impact, LLC, Battle Creek, MI, United States; 3 NutriScience, LLC, East Norriton, PA, United States

**Keywords:** beverages, calcium, children, magnesium, milk, National Health and Nutrition Examination Survey, NHANES, protein, SSB, vitamin D

## Abstract

**Background:**

Milk is a key source of important nutrients including the nutrients of public health concern. However, most Americans do not meet current (dairy) United States Department of Agriculture (USDA) dietary guideline recommendations, and the intake has been declining.

**Objective:**

The aim of this study was to investigate milk and beverage intake trends and nutrient intakes from these products in United States children aged 6–18 y and to model the effect of isocaloric substitution of nondairy beverages with milk.

**Methods:**

Data from National Health and Nutrition Examination Survey (NHANES) 2001–2018 for children age 6–8 (*N* = 4696), 9–13 (*N* = 8117) and 14–18 y (*N* = 8514) were used with milk and other beverage intakes determined from the first 24-h in-person dietary recall. Nutrient intake was determined using the NHANES cycle-specific total nutrient intake files. Nutrient modeling was performed by isocaloric substitution with milk of all nondairy beverages consumed during lunch and dinner meals combined. Sample-weighted analyses were performed using SAS 9.4.

**Results:**

Between ages 6–8 and 14–18 y, daily intake of milk and flavored milk decreased by 10% and 62%, respectively, while daily intake of caloric beverages excluding milk increased by 96%. Daily intake from caloric beverages and milk combined decreased for fiber, protein, fat, saturated fat, calcium, magnesium, potassium, vitamin A, and vitamin D and increased for energy, carbohydrates, added sugars, and folate between ages 6–8 y and 14–18 y. Isocaloric substitution of all caloric nondairy beverages at meals with milk (using nutrient contribution of USDA milk, not further specified (NFS)) resulted in increases in protein, fat, saturated fat, calcium, magnesium, potassium, sodium, vitamin A, folate, vitamin B_12_, and vitamin D and decreases in carbohydrate, fiber, and added sugar.

**Conclusion:**

These findings provide additional evidence to support dietary recommendations for milk, and efforts should be made on behalf of leading health professionals and childhood meal programs to highlight milk as a beverage of choice in children and adolescents.

## Introduction

The Dietary Guidelines for Americans are intended to promote the intake of a variety of food groups, including dairy, and provide dietary advice to help meet nutrient needs, promote health, and reduce risk of disease across all stages of life. The current version recommends 2.5 daily servings (cup equivalents) of dairy products for children aged 4–8 y and 3 daily servings for those aged 9–18 y to ensure that they meet nutrient intake recommendations, promote healthy growth and development, as well as reduce risk of chronic diseases [1].

Cow milk is a key source of high-quality protein and essential micronutrients, such as calcium, phosphorus, vitamin A, vitamin D, riboflavin, niacin, pantothenic acid, vitamin B_12_, iodine, potassium, selenium, and zinc [2] and contributes to overall diet quality in both children and adults [1]. Calcium, potassium, dietary fiber, and vitamin D are underconsumed in the general United States population and have been identified as “nutrients of public health concern” because low intakes are associated with health concerns [1]. Additionally, children and adolescents underconsume phosphorus, magnesium, and choline among both male and females and protein, iron, folate, vitamin B_6_, and vitamin B_12_ among females [3]. Data suggest that children and adolescents who meet dairy recommendations are less likely to be below recommended levels (Estimated Average Requirements or Adequate Intakes) of several essential nutrients, including calcium, magnesium, phosphorus, riboflavin, vitamin A, vitamin B_12_, vitamin D, selenium, potassium, and choline [4]. Moreover, milk was reported to be the leading food source of 3 of the 4 nutrients of public health concern, calcium, vitamin D, and potassium, providing 22%–23%, 46%–47%, and 12%–13%, respectively, of daily intake for children aged 6–18 y [5].

Approximately 90% of Americans do not meet the recommendations for dairy intake, and only ∼65% of children, 34% of adolescents, and 20% of adults drink milk on a given day [1]. Additionally, intakes of dairy and particularly fluid milk have been slowly but steadily decreasing in the United States as well as globally [[6], [7], [8], [9], [10]]. Daily per capita consumption of fluid cow milk has declined by approximately 50% from approximately a cup in 1970 to about half cup in 2019 [6,7]. Although sugar-sweetened beverages (SSBs), such as soft drinks and juice drinks appeared to be replacing milk in earlier years, the current increase in the number of caloric beverages commercially available, such as energy drinks, or more recently, the proliferation of plant-based milk alternatives may help to explain this trend; however, the extent of this impact is yet to be determined [6,7].

The current study investigated the current trends in fluid milk and beverage intake as well as nutrient status in United States children aged 6–18 y using the NHANES, a nationally representative, continuous survey of the noninstitutionalized civilian United States population [11]. We hypothesize that as milk consumption declines in this age group, consumption of less nutrient-dense beverages will rise, resulting in a drop of certain essential nutrients in the overall diet and a reduction of nutrient adequacy and overall diet quality. We further hypothesize that adding 1 serving of milk per day or isocaloric substitution of all caloric beverages consumed at meals with milk will result in significant improvement in overall nutritional intake and consequently nutrient adequacy within this age group.

## Methods

### Data source and subjects

What We Eat in America (WWEIA) is the nutritional component of NHANES that collects 2 24-h dietary recalls from its subjects using the automated multiple-pass method, which includes multiple passes to increase recall accuracy [12]. The present analysis combined 9 NHANES datasets [11] over 2 decades (NHANES 2001–2002, 2003–2004, 2005–2006, 2007–2008, 2009–2010, 2011–2012, 2013–2014, 2015–2016, and 2017–2018). The combined sample included 21,327 children aged 6–18 y after excluding subjects who were pregnant and/or lactating (*n* = 116) and those with incomplete or unreliable dietary recalls as determined by USDA (*n* = 5475).

### Milk, beverages, and nutrient intake

The milk and beverage intakes were estimated from the first 24-h in-person dietary recall. Data were collected by proxy (the person with the most knowledge of their food intake)-assisted interviews of children aged 6–11 y and individual interviews for those aged ≥12 y, as per NHANES dietary interview protocols. Details of NHANES protocols are available in previously published literature [11,13]. WWEIA food categories were used to define milk and other beverages: milk (USDA subgroup 10), flavored milk (USDA subgroup 12), milk substitutes (USDA category 1404), fruit juice (USDA subgroup 70), SSBs (USDA subgroup 72), soft drinks (USDA category 7202), fruit drinks (USDA category 7204), coffee and tea (USDA subgroup 73), caloric beverages, excluding milk (USDA main group 8 and subgroups not in 71, 77, 78), and caloric beverages, including milk (also included milk, flavored milk, and milk substitutes) [14]. Nutrient and energy intake was determined using the NHANES cycle-specific total nutrient intake files based on Food Nutrient Database for Dietary Studies, which provides nutrient content for food and beverages reported in WWEIA [14].

### Demographic variables

Demographic variables included in the analysis were age, sex (% male), race/ethnicity (Mexican American, other Hispanic, non-Hispanic White, non-Hispanic Black, and Other), poverty–income ratio (PIR; <1.35, 1.35 to ≤1.85, and >1.85), physical activity level (sedentary, moderate, and vigorous), and weight status (underweight, normal weight, overweight, and obese). Physical activity level categories were based on the NHANES physical activity questionnaire. Weight status was defined as based on BMI *z*-score of <5% (underweight), 5%–<84.9% (normal weight), 85%–94.9% (overweight), ≥95% (obese) using the Centers for Disease Control and Prevention weight-for-age program [15].

### Nutrient modeling

The following 2 modeling scenarios were utilized to assess changes in nutrient intake: *1*) 1 serving of milk was added to the daily diet of each subject using nutrients from 1 cup of milk (NHANES food code 11100000; milk, nfs) (nfs, not further specified, which is a combination of various fat levels of milk); and *2*) an isocaloric substitution of all nonmilk caloric beverages consumed at meals with milk where nutrients from all caloric beverages (SSBs, milk substitutes, or all beverages in WWEIA beverage subgroup) consumed during lunch and dinner meals combined were substituted with nutrients from isocaloric amount of milk using nutrient profile of NHANES food code 11100000 milk, nfs. Nutrient contents of milk (NHANES food code 11100000; milk, nfs) are provided in Supplemental Table 1.

### Statistical analyses

All analyses were performed using SAS 9.4 (SAS Institute) using PROC SURVEYMEANS, and survey parameters, including primary sampling unit, strata, and day 1 dietary weights were used to account for NHANES’ complex sample design and to provide nationally representative estimates using Taylor series expansion to generate variance estimates. The nutritional contribution of caloric beverages was assessed using the recommended population ratio method. Regression analyses were used to assess changes in intake across the age range (6–18 y) using PROC SURVEYREG; the regression coefficient generated from these analyses represented the change across age in change per year. Sex, ethnicity, and PIR level were used as covariates. Although some analyses were conducted across the entire age group (i.e., regression analyses examining changes across age and nutrition contribution of caloric beverages), other analyses were also conducted for separate age groups (6–8, 9–13, and 14–18 y) to allow comparisons with previous published results. Because typical statistical testing was not appropriate for modeling of adding a serving milk to all subjects (given violation of assumption of independent samples), we set a 10% change in intake as a meaningful difference. For replacement of caloric beverages at meals, paired *t* tests were used to assess changes in intakes.

## Results

### Demographics

Across the 3 age groups, ∼50% to 52% of children were male, and the majority were non-Hispanic White (56%–59%), followed by non-Hispanic Black (14%), Mexican American (14%), other (8%–8.5%), and other Hispanic (6%–7%) (Table 1). Over 50% were in the higher PIR (PIR >1.85) category, and a third were in the lowest PIR (PIR <1.35) category. Approximately 58%–66% reported having a vigorous physical activity level, and 60%–64% were of normal weight (Table 1).

**TABLE 1:** Demographics of children by age groups, NHANES 2001–2018

Variables	Age 6–8 y	Age 9–13 y	Age 14–18 y
*N*	4696	8117	8514
Age (mean)	7.01 ± 0.02	11.0 ± 0.02	16.0 ± 0.02
Male (%)	52.3 ± 1.1	50.2 ± 0.9	50.6 ± 0.8
Ethnicity (%)			
Mexican American	14.5 ± 1.1	14.1 ± 1.0	13.4 ± 1.0
Other Hispanic	6.70 ± 0.64	6.78 ± 0.62	5.96 ± 0.55
Non-Hispanic White	56.4 ± 1.9	56.3 ± 1.6	58.8 ± 1.5
Non-Hispanic Black	14.1 ± 1.0	14.4 ± 0.9	14.0 ± 0.9
Other	8.29 ± 0.62	8.47 ± 0.59	7.76 ± 0.53
Poverty–income ratio (%)			
<1.35	34.8 ± 1.3	32.4 ± 1.2	31.4 ± 1.1
1.35 to ≤1.85	11.2 ± 0.8	11.5 ± 0.6	10.2 ± 0.7
>1.85	54.0 ± 1.6	56.1 ± 1.3	58.4 ± 1.2
Physical activity (%)			
Sedentary	14.3 ± 0.8	16.1 ± 0.7	12.8 ± 0.6)
Moderate	19.9 ± 1.0	25.5 ± 0.7	23.9 ± 0.8
Vigorous	65.7 ± 1.3	58.4 ± 0.9	63.3 ± 1.0
Weight status (%)			
Underweight	3.73 ± 0.59	3.60 ± 0.35	3.17 ± 0.36
Normal weight	64.1 ± 1.2	60.2 ± 0.8	61.7 ± 0.9
Overweight	14.8 ± 0.8	16.9 ± 0.6	16.2 ± 0.6
Obese	17.4 ± 0.9	19.4 ± 0.8	18.9 ± 0.8

Data presented as mean ± SEM.

### Food group intake

Daily intakes of milk and flavored milk decreased by 9.6% (210–190 g); the change in intake in g/d/y of age (regression coefficient for linear trend: β_linear trend_) was −2.64, (*P* = 0.0010) and 62% (91.7–34.9 g; β_linear trend_ = −6.21, *P* < 0.0001), respectively, in those 6–8 y compared with those 14–18 y. Daily intakes of other beverages increased between ages 6–8 y and 14–18 y by 96% (β_linear trend_ = 43.7, *P* < 0.0001) for caloric beverages excluding milk, 185% (β_linear trend_ = 25.3, *P* < 0.0001) for soft drinks, 103% (β_linear trend_ = 30.8 g, *P* < 0.0001) for SSBs, and 332% (β_linear trend_ = 13.1 g, *P* < 0.0001) for coffee and teas. However daily intakes of milk substitutes, 100% fruit juice, and fruit drinks did not change (*P* > 0.05) across age groups (Table 2).

**TABLE 2:** Mean intake of beverages among children of different age groups, sex-combined data NHANES 2001–2018

	Age 6–8 y	Age 9–13 y	Age 14–18 y	Regression coefficient	*P*_Linear Trend_
Energy (kcal)	1869 ± 13	2029 ± 14	2209 ± 18	35.9 ± 2.5	<0.0001
Milk (g)	210 ± 5	208 ± 6	190 ± 6	-2.64 ± 0.79	0.0010
Flavored milk (g)	91.7 ± 4.6	61.1 ± 2.6	34.9 ± 2.5	-6.21 ± 0.45	<0.0001
Milk substitutes (g)	3.83 ± 0.86	2.23 ± 0.61	3.18 ± 0.61	-0.01 ± 0.11	0.8962
Caloric beverages including milk (g)	702 ± 11	782 ± 12	1004 ± 15	34.8 ± 2.1	<0.0001
Caloric beverages excluding milk (g)	396 ± 9	511 ± 11	776 ±15	43.7 ± 2.1	<0.0001
100% juice (g)	94.4 ± 4.1	76.5 ± 2.6	84.9 ± 4.3	-0.23 ± 0.66	0.7334
Soft drinks (g)	123 ± 6	211 ± 8	350 ± 12	25.3 ± 1.4	<0.0001
SSB (g)	268 ± 8	370 ± 10	546 ± 13	30.8 ± 1.6	<0.0001
Fruit drinks (g)	125 ± 4	123 ± 4	136 ± 5	1.42 ± 0.72	0.0507
Coffee, tea (g)	33.6 ± 3.5	64.1 ± 4.2	145 ± 9	13.1 ± 1.2	<0.0001

Data presented as least square mean ± SEM after adjusting for sex, ethnicity, and poverty–income ratio level. Regression coefficient represents the change across ages 6 to 18 y and indicates the change per 1 y of age. Abbreviation: SSB, sugar-sweetened beverage.

### Nutrient contribution of the caloric beverages

**FIGURE 1:**
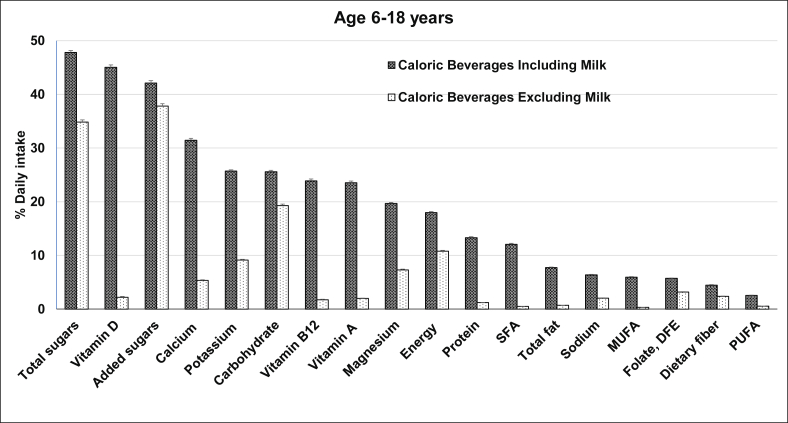
Contribution of caloric beverages to daily intake of nutrients among children age 6–18 y (*N* = 21,327) gender-combined data NHANES 2001–2018. Data presented as mean and SEM. DFE, dietary folate equivalent; MUFA, monounsaturated fatty acid; PUFA, polyunsaturated fatty acid; SFA, saturated fatty acid.

Nutrient contributions of the caloric beverages including and excluding milk to daily intakes of children aged 6–18 y are presented in Figure 1. As expected, nutrient contributions of caloric beverages including milk were more than that of caloric beverages without milk. Caloric beverages, including milk, contributed 18% of total calories, and more than half (11%) was provided by caloric beverages excluding milk. Caloric beverages excluding milk contributed to 1%–2% of daily intakes of protein, vitamin A, vitamin B_12_, vitamin D and 5% calcium while caloric beverages, including milk contributed to 13% protein, 24% vitamin A, 24% vitamin B_12_, 45% vitamin D, and 31% calcium. Total fat and saturated fat were mainly contributed by caloric beverages including milk (8% and 12%, respectively) while caloric beverages excluding milk contributed <1% total fat and saturated fat. Caloric beverages including or excluding milk contributed comparable amounts (42% compared with 38%, respectively) of added sugars (Figure 1).

### Nutrient intake from caloric beverages including milk

Daily intake of nutrients from caloric beverages including milk decreased with age and percentage decreases between ages 6 to 8 y and 14 to 18 y in g/d/y of age were: 30% for fiber (β_linear trend_ = −0.02, *P* < 0.0001), 19% for protein (β_linear trend_ = −0.22, *P* < 0.0001), 23% for fat (β_linear trend_ = −0.15 g, *P* < 0.0001), 24% for MUFAs (β_linear trend_ = −0.04 g, *P* < 0.0001), 20% for PUFAs (β_linear trend_ = −0.01 g, *P* < 0.0001), 25% for saturated fatty acids (SFAs; β_linear trend_ = −0.11 g, *P* < 0.0001), 20% for calcium (β_linear trend_ = −9.09 mg, *P* < 0.0001), 6% for magnesium (β_linear trend_ = −0.24 mg, *P* = 0.0368), 11% for potassium (β_linear trend_ = −6.82 mg, *P* < 0.0001), 21% for vitamin A (β_linear trend_ = −3.91 μg retinol equivalents, *P* < 0.0001), and 24% for vitamin D (β_linear trend_ = −0.10 μg, *P* < 0.0001).The daily intake increased 20% for energy (β_linear trend_ = 8.00 kcal, *P* < 0.0001), 28% for carbohydrates (β_linear trend_ = 2.67 g, *P* < 0.0001), 39% for total sugar (β_linear trend_ = 2.51 g, *P* < 0.0001), 88% for added sugar (β_linear trend_ = 0.70 teaspoon, *P* < 0.0001), and 12% for folate (β_linear trend_ = 0.42 μg dietary folate equivalents, *P* < 0.0001) (Table 3).

**TABLE 3:** Mean intake of nutrients from caloric beverages including milk among children of different age groups, sex-combined data NHANES 2001–2018

Nutrient	Age 6–8 y	Age 9–13 y	Age 14–18 y	Regression coefficient	*P*_Linear Trend_
Energy (kcal)	335 ± 5	343 ± 5	402 ± 6	8.00 ± 0.87	<0.0001
Carbohydrate (g)	60.7 ±1.1	65.8 ± 1.1	83.5 ± 1.4	2.67 ± 0.20	<0.0001
Dietary fiber (g)	0.74 ± 0.03	0.57 ± 0.02	0.52 ± 0.02	-0.02 ± 0.004	<0.0001
Total sugars (g)	56.1 ± 1.0	61.3 ± 1.0	77.8 ± 1.3	2.51 ± 0.19	<0.0001
Added sugars (tsp eq)	7.04 ± 0.17	9.11 ± 0.21	13.2 ± 0.3	0.70 ± 0.04	<0.0001
Protein (g)	10.4 ± 0.2	9.46 ± 0.22	8.44 ± 0.20	-0.22 ± 0.03	<0.0001
Total fat (g)	6.17 ± 0.15	5.35 ± 0.14	4.78 ± 0.13	-0.15 ± 0.02	<0.0001
Total MUFA (g)	1.60 ± 0.04	1.39 ± 0.04	1.22 ± 0.04	-0.04 ± 0.005	<0.0001
Total PUFA (g)	0.35 ± 0.01	0.29 ± 0.01	0.28 ± 0.01	-0.01 ± 0.001	<0.0001
Total SFA (g)	3.62 ± 0.09	3.12 ± 0.09	2.68 ± 0.08	-0.11 ± 0.01	<0.0001
Calcium (mg)	408 ± 8	370 ± 9	326 ± 7	-9.09 ± 1.07	<0.0001
Magnesium (mg)	50.6 ± 0.9	46.6 ± 0.9	47.7 ± 0.9	-0.24 ± 0.11	0.0368
Potassium (mg)	658 ± 13	596 ± 12	587 ± 11	-6.82 ± 1.60	<0.0001
Sodium (mg)	190 ± 4	183 ± 4	185 ± 4	-0.63 ± 0.50	0.2113
Vitamin A, RE (μg)	169 ± 4	153 ± 4	134 ± 3	-3.91 ± 0.48	<0.0001
Folate, DFE (μg)	26.0 ± 0.8	25.5 ± 0.7	29.0 ± 0.9	0.42 ± 0.12	0.0006
Vitamin B_12_ (μg)	1.40 ± 0.03	1.31 ± 0.03	1.38 ± 0.05	0.002 ± 0.01	0.8124
Vitamin D (μg)	3.76 ± 0.08	3.36 ± 0.08	2.87 ± 0.08	-0.10 ± 0.01	<0.0001

Data presented as least square mean ± SEM after adjusting for sex, ethnicity, and poverty–income ratio level. Regression coefficient represents the change across ages 6 to 18 y and indicates the change per 1 y of age.

Abbreviations: DFE, dietary folate equivalent; MUFA, monounsaturated fatty acid; PUFA, polyunsaturated fatty acid; RE, retinol equivalent; SFA, saturated fatty acid; tsp eq, teaspoon equivalent.

### Modeling nutrient intake with addition of milk

With the addition of 1 cup of milk (NHANES food code 11100000 milk, nfs), intake of several nutrients increased including calcium (28%–29%), magnesium (11%–12%), potassium (15%–17%), protein (10%–13%), SFAs (10%–12%), total sugars (9%–10%), and vitamins A (20%–22%), B_12_ (21%–24%), and D (51%–58%). Carbohydrates, fiber, folate, energy, MUFAs, PUFAs, sodium, and total fat increased <10% with the addition of a serving of milk (Table 4).

**TABLE 4:** Mean intake of nutrients among children of different age groups after addition of 1 cup eq of milk, sex-combined data NHANES 2001–2018

Nutrient	Age 6–8 y		Age 9–13 y		Age 14–18 y	
	Baseline	Adjusted	Baseline	Adjusted	Baseline	Adjusted
Energy (kcal)	1869 ± 13	1991 ± 13	2028 ± 14	2150 ± 14	2209 ± 18	2331 ± 18
Carbohydrate (g)	253 ± 2	265 ± 2	270 ± 2	282 ± 2	288 ± 2	300 ± 2
Dietary fiber (g)	13.1 ± 0.1	13.1 ± 0.1	14.3 ± 0.2	14.3 ± 0.2	14.3 ± 0.1	14.3 ± 0.1
Total sugars (g)	125 ± 1	137 ± 1	129 ± 1	142 ± 1	140 ± 1	153 ± 1
Added sugars (tsp eq)	18.2 ± 0.2	18.2 ± 0.2	20.3 ± 0.3	20.3 ± 0.3	23.6 ± 0.3	23.6 ± 0.3
Protein (g)	63.3 ± 0.5	71.4 ± 0.5	71.1 ± 0.7	79.2 ± 0.7	79.8 ± 0.8	87.8 ± 0.8
Total fat (g)	69.6 ± 0.6	74.4 ± 0.6	76.4 ± 0.6	81.2 ± 0.6	83.3 ± 0.8	88.1 ± 0.8
Total MUFA (g)	24.5 ± 0.3	25.8 ± 0.3	27.0 ± 0.2	28.3 ± 0.2	29.7 ± 0.3	31.0 ± 0.3
Total PUFA (g)	14.3 ± 0.2	14.6 ± 0.2	16.0 ± 0.2	16.2 ± 0.2	17.9 ± 0.2	18.1 ± 0.2
Total SFA (g)	24.7 ± 0.2	27.6 ± 0.2	26.8 ± 0.3	29.7 ± 0.3	28.4 ± 0.3	31.3 ± 0.3
Calcium (mg)	1000 ± 11	1291 ± 11	1024 ± 13	1314 ± 13	1032 ± 12	1322 ± 12
Magnesium (mg)	220 ± 2	247 ± 2	237 ± 2	265 ± 2	255 ± 2	282 ± 2
Potassium (mg)	2095 ± 21	2453 ± 21	2207 ± 21	2565 ± 21	2349 ± 25	2707 ± 25
Sodium (mg)	2882 ± 26	2985 ± 26	3244 ± 29	3347 ± 29	3596 ± 38	3699 ± 38
Vitamin A, RE (μg)	603 ± 10	727 ± 10	613 ± 10	737 ± 10	573 ± 8	696 ± 8
Folate, DFE (μg)	514 ± 8	525 ± 8	546 ± 7	558 ± 7	564 ± 8	575 ± 8
Vitamin B_12_ (μg)	4.68 ± 0.07	5.82 ± 0.07	4.97 ± 0.08	6.11 ± 0.08	5.36 ± 0.08	6.50 ± 0.08
Vitamin D (μg)	5.82 ± 0.09	8.81 ± 0.09	5.55 ± 0.10	8.54 ± 0.10	5.19 ± 0.09	8.18 ± 0.09

Data presented as least square mean ± SEM after adjusting for sex, ethnicity, and poverty–income ratio level.

Abbreviations: DFE, dietary folate equivalent; MUFA, monounsaturated fatty acid; PUFA, polyunsaturated fatty acid; RE, retinol equivalent; SFA, saturated fatty acid; tsp eq, teaspoon equivalent.

### Modeling nutrient intake after isocaloric replacement of caloric beverages at meals

With the isocaloric replacement of caloric beverages with milk (NHANES food code 11100000 milk, nfs) at lunch and dinner (∼0.4–0.5 cups across age groups), several nutrients increased (*P* < 0.01 by paired *t* test) including protein (5%–6%), fat (3%), MUFAs (2%), PUFAs (1%), SFAs (5%–6%), calcium (12%–14%), magnesium (4%), potassium (5%–6%), sodium (1%), vitamin A (9%–11%), folate (1%), vitamin B_12_ (11%), and vitamin D (23%–31%); there were decreases (*P* < 0.01 by paired *t* test) in carbohydrate (3%), fiber (1%), total sugar (5%–6%), and added sugar (13%–14%) (Table 5).

**TABLE 5:** Mean intake of nutrients among children of different age groups after isocaloric replacement of all caloric beverages during lunch and dinner with milk, sex-combined data NHANES 2001–2018

Nutrient	Age 6–8 y		Age 9–13 y		Age 14–18 y	
	Baseline	Adjusted	Baseline	Adjusted	Baseline	Adjusted
Energy (kcal)	1869 ± 13	1869 ± 13	2028 ± 14	2028 ± 14	2209 ± 18	2209 ± 18
Carbohydrate (g)	253 ± 2	245 ± 2∗	270 ± 2	261 ± 2∗	288 ± 2	278 ± 2∗
Dietary fiber (g)	13.1 ± 0.1	13 ± 0.1∗	14.3 ± 0.2	14.2 ± 0.2∗	14.3 ± 0.1	14.2 ± 0.1∗
Total sugars (g)	125 ± 1	118 ± 1∗	129 ± 1	122 ± 1∗	140 ± 1	132 ± 1∗
Added sugars (tsp eq)	18.2 ± 0.2	15.9 ± 0.2∗	20.3 ± 0.3	17.5 ± 0.2∗	23.6 ± 0.3	20.4 ± 0.3∗
Protein (g)	63.3 ± 0.5	66.9 ± 0.5∗	71.1 ± 0.7	75 ± 0.7∗	79.8 ± 0.8	83.9 ± 0.8∗
Total fat (g)	69.6 ± 0.6	71.7 ± 0.6∗	76.4 ± 0.6	78.7 ± 0.6∗	83.3 ± 0.8	85.8 ± 0.8∗
Total MUFA (g)	24.5 ± 0.3	25.1 ± 0.3∗	27.0 ± 0.2	27.6 ± 0.2∗	29.7 ± 0.3	30.4 ± 0.3∗
Total PUFA (g)	14.3 ± 0.2	14.4 ± 0.2∗	16.0 ± 0.2	16.1 ± 0.2∗	17.9 ± 0.2	18 ± 0.2∗
Total SFA (g)	24.7 ± 0.2	26 ± 0.2∗	26.8 ± 0.3	28.3 ± 0.3∗	28.4 ± 0.3	30 ± 0.3∗
Calcium (mg)	1000 ± 11	1120 ± 11∗	1024 ± 13	1157 ± 13∗	1032 ± 12	1178 ± 12∗
Magnesium (mg)	220 ± 2	228 ± 2∗	237 ± 2	247 ± 2∗	255 ± 2	265 ± 2∗
Potassium (mg)	2095 ± 21	2203 ± 20∗	2207 ± 21	2339 ± 20∗	2349 ± 25	2494 ± 24∗
Sodium (mg)	2882 ± 26	2915 ± 26∗	3244 ± 29	3280 ± 29∗	3596 ± 38	3635 ± 38∗
Vitamin A, RE (μg)	603 ± 10	657 ± 10∗	613 ± 10	672 ± 10∗	573 ± 8	638 ± 8∗
Folate, DFE (μg)	514 ± 8	516 ± 7∗	546 ± 7	549 ± 7∗	564 ± 8	567 ± 8∗
Vitamin B_12_ (μg)	4.68 ± 0.07	5.19 ± 0.07∗	4.97 ± 0.08	5.52 ± 0.08∗	5.36 ± 0.08	5.93 ± 0.08∗
Vitamin D (μg)	5.82 ± 0.09	7.17 ± 0.09∗	5.55 ± 0.10	7.02 ± 0.10∗	5.19 ± 0.09	6.78 ± 0.09∗

Data presented as least square mean ± SEM after adjusting for gender, ethnicity and and poverty-income-ratio level.

Abbreviations: DFE, dietary folate equivalent; MUFA, monounsaturated fatty acid; PUFA, polyunsaturated fatty acid; RE, retinol equivalent; SFA, saturated fatty acid; tsp eq, teaspoon equivalent. ∗Different from baseline at *P* < 0.01 by paired *t* test.

## Discussion

The results of the present analysis indicate that with increasing age, i.e., moving from early childhood to teenage years, the intake of milk decreased whereas the intakes of soft drinks and SSBs increased and intakes of milk substitutes, 100% juice, and fruit drinks did not change. Consequently, the intakes of most nutrients from all caloric beverages, including milk decreased with age whereas the intake of energy and sugars (including added sugar) increased with age. The addition of a serving of milk to the daily diet or isocaloric replacement of all caloric beverages at lunch and dinner with milk resulted in an increase in several micronutrients including calcium, vitamin A, vitamin B_12_, and vitamin D with only a 1–3 g/d increase in saturated fat across both modeling scenarios and a 2–3 teaspoon equivalent/d (8–12 g/d) decrease in added sugars for replacing caloric beverages at meals.

Milk is an important source of nutrients, and the consumption of milk by children has been positively correlated with diet quality as well as meeting nutrient recommendations, including vitamin A, folate, vitamin B_12_, calcium, and magnesium in the United States [5,16] as well as globally [[17], [18], [19], [20], [21]]. In our analysis, beverages, including milk, provided substantial amounts (>20% of daily intake) of vitamins and minerals while the contribution of beverages excluding milk was minimal (≤5%) to daily intakes of these vitamins and minerals. This suggests that nutrient density (nutrient to energy ratio) of milk is much more than that of other caloric beverages. In the present analysis, the intake of milk decreased and the intakes of soft drinks and SSB increased with age, whereas the intakes of most nutrients (from caloric beverages including milk) decreased, and energy and added sugar increased.

SSBs, by definition, are beverages containing added sugar and/or sweetener and include regular soda, fruit drinks, sports drinks, energy drinks, presweetened ready-to-drink tea, and sweetened ready-to-drink coffee. SSB consumption begins during the preschool years and generally increases with age [22,23]. In our analysis, we also found that intake of SSBs, including soda and fruit drinks, increased with age with a concomitant decrease in milk intake. These beverage consumption changes have been of public health concern due to their suspected linkage with health-related factors, such as excess energy intake, overweight and obesity, poor cardiometabolic health, and insulin resistance [[24], [25], [26], [27]]. Additionally, SSB consumption by young children and adolescents has been linked to reduced milk intake and negatively correlated with achieving intakes of vitamin A, vitamin C, and calcium [[28], [29], [30]]. A number of studies have examined the impact of swapping various beverages with milk. In a randomized crossover study, a 3-wk intervention with milk replacing soda decreased systolic blood pressure, uric acid, and glycosphingolipids but did not affect lipoproteins in male adolescents [31]. In a systematic review and meta-analysis of randomized control trials, substituting SSBs with flavored milk or noncaloric beverages significantly reduced body fat, with no change in BMI [32]. In a European cohort of children, replacement of SSBs with water or milk was associated with reduced body fatness [33]. In another study, although replacing habitual consumption of SSBs with milk did not affect the percent body fat, lean body mass was significantly increased [34]. Isocaloric replacement of all caloric beverages (including soft drinks and SSBs) with milk at lunch and dinner was found to increase intake of protein and several key micronutrients in the present modeling analysis.

Flavored milk is prepared from dairy milk and edible flavorings with or without caloric sweeteners. There is a perception by some that consumption of flavored milk will have a negative impact on the overall quality of children’s diets due to added sugar, and some have called for banning flavored milk from school lunchrooms [[35], [36], [37]]. However, flavored milk is a nutrient-rich beverage and has a nutrient profile similar to that of plain milk. Flavored milk is a rich source of calcium, protein, vitamin D, vitamin A, vitamin B_12_, potassium, phosphorus, riboflavin, and niacin. Although sweetened flavored milk has added sugar, in the United States, the contribution of added sugar from flavored milk is only ∼3% of total energy [38] and is much less than that from SSBs [39]. A review of scientific studies concluded that flavored milk consumption was not associated with higher added sugar intake [40]. Flavored milk is a palatable beverage choice that helps children to meet their nutrient needs, and children who consumed flavored milk were found to consume more total milk and had higher intakes of nutrients, specifically calcium, phosphorus, magnesium, potassium, and vitamins A and D in multiple scientific studies [16,17,38,[40], [41], [42], [43]]. Acceptability of flavored milk, especially chocolate milk, among children is much higher than that of plain milk, and studies suggest that children choose flavored over plain milk and consume ∼30% more total milk [[44]; M. Read, K. Henderson, M. Schwartz, unpublished results, 2011]. Additionally, in a modeling study [45], removal of flavored milk from school meals resulted in decreased milk consumption and increased milk waste, and to make up for lost nutrients, 3–4 additional foods (including cheese, baked beans, vegetables, and fruit yogurt) were consumed with additional fat and calories at an increased dollar amount (∼$50 per student per year). In the present study, flavored milk intake was highest among children aged 6–8 y and consistently decreased with age, whereas children aged 14–18 y consumed ∼62% less flavored milk than younger children. Encouraging older adolescents to replace SSBs with flavored milk could benefit nutrient intake and lessen the gap between current dairy intake recommendations.

Milk substitutes, including almond milk, soy milk, rice milk, oat milk, and coconut milk, are nondairy beverages made from plant-based ingredients (such as rice, nuts/seeds, coconut, oats, peas, or blends of these ingredients). They are available in various flavors and options such as plain, vanilla, or chocolate, as well as organic, fortified or unfortified, and sweetened (with added sugar) or unsweetened. They have become popular over the past 2 decades; however, they are often a nutritionally inferior substitute to dairy milk with the exception of fortified soy, and scientists have advocated for nutritional standards for these beverages [46,47]. In the present analysis, intake of these milk substitutes was very small (<5 g/d) and was not affected by age.

Dairy (including milk, cheese, and yogurt) is an important food group and an important component of a healthy diet and is included in the Healthy Dietary Patterns developed by USDA and released as part of Dietary Guidelines for Americans 2020–2025 [1] and in MyPlate recommendations [48]. Despite these recommendations, a vast majority of Americans (>80%) consume less than the recommended intake of dairy [1]. Although adequate dairy intake is associated with reduced risk of various noncommunicable chronic diseases [49], lower dairy intake among children and adolescents not only affects their growth and development but impacts their adult life. A study published in 2012 reported that childhood milk intake was positively associated with physical performance in old age [50]. There is an increasing body of evidence to suggest that dairy consumption, including total dairy and fuller fat milk, may be beneficial to overall nutrient status and cardiometabolic health [32,51,52]. Intake of dairy products including milk has been associated with reduced prevalence and risk of obesity among children [32,[52], [53], [54], [55]] and is beneficial for reducing the risk of a variety of chronic diseases in adulthood [51,56]. However, beverage milk consumption has been consistently declining since 1975 [57]. Additionally, due to the ongoing debate about the environmental impact from animal agriculture, some are advocating to remove or limit animal-sourced foods, including dairy [58,59]. However, limiting relatively low-cost nutrient-dense foods, such as milk or substituting milk with other caloric beverages, such as soft drinks and SSBs could have potential unintended consequences, such as increased nutrient inadequacy and/or deficiencies [[60], [61], [62]].

Strengths of this study include the use of large nationally representative sample achieved through combining several sets of NHANES data releases and the same-person modeling used in this study. A major limitation of this study is the use of self-reported dietary intake data, which therefore are subject to reporting bias [63]. Additionally, the dietary modeling approaches used here are focused on evaluating the maximum effect (assuming all subjects would comply with a modeled condition), may not reflect actual individual dietary behavior, and can only be used as an estimate of potential impact to nutrient intake as they cannot predict actual compliance in free-living individuals; however, such modeling offers a technique to test the potential nutritional impact of dietary guidance. Future directions should include modeling the replacement of all caloric beverages at mealtimes with commonly chosen plant-based milk alternatives to better understand the impact of mealtime choices and provide more informed guidance.

In conclusion, intake of SSBs, including soft drinks, other caloric beverages, and added sugar, as well as energy, increased with age whereas intake of milk decreased and consequently, the nutrient contribution of caloric beverages including milk also decreased. Additionally, the results of this study show that the addition of a serving of milk or isocaloric replacement of beverages with milk resulted in an increase in several micronutrients, including calcium, potassium, vitamin D, magnesium, and protein and no change or decrease in added sugar. The current findings provide additional evidence to support dietary recommendations for milk, and increased efforts appear to be needed to reverse the decrease in milk intake over time and as children age.

## Author contributions

The authors’ contributions were as follows – KR-J, MAP, CJC: participated in the formulation of the research question, design of the analyses, revision of the manuscript; KF: participated in the revision of the manuscript; VLF: participated in the formulation of the research question, design of analyses, NHANES dietary data analysis, interpretation of the data, revision of the manuscript; SA: participated in the interpretation of the data, drafting of the manuscript, revision of the manuscript; and all authors: read and approved the final manuscript and are responsible for all aspects of the manuscript.

## Funding

The study and the writing of the manuscript were supported by National Dairy Council.

## Data availability

The datasets analyzed in this study are available in the Centers for Disease Control and Prevention repository, available online at: http://www.cdc.gov/nchs/nhanes/ [cited 6 June, 2022]. Upon request, authors are willing to help others to replicate or conduct similar analyses.

## Declaration of interests

The authors declare the following financial interests/personal relationships which may be considered as potential competing interests: Kristin Ricklefs-Johnson reports financial support was provided by National Dairy Council. Kristin Ricklefs-Johnson reports a relationship with National Dairy Council that includes: employment. K.R.J., M.A.P. and C.J.C. work for the National Dairy Council. V.L.F. III and K.F. work for Nutrition Impact LLC performs consulting and database analyses for various food and beverage companies and related entities including the National Dairy Council. S.A. as Principal of NutriScience LLC performs nutrition science consulting for various food and beverage companies and related entities.

## Conflict of interest

KR-J, MAP, and CJC are employees of the National Dairy Council, Rosemont, IL, United States. KF and VLF at Nutrition Impact, LLC perform consulting and database analyses for various food and beverage companies and related entities. SA at NutriScience, LLC performs consulting for various food and beverage companies and related entities.
