# Supplementary material for: Assessment of Beverage Trends and Replacing Nondairy Caloric Beverages with Milk at Meals across Childhood Improves Intake of Key Nutrients at Risk of Inadequate Consumption: An NHANES Modeling Study
Source: Curr Dev Nutr. 2023 Oct 18;7(11):102020. doi: 10.1016/j.cdnut.2023.102020 (PMC10681945; doi:10.1016/j.cdnut.2023.102020)
Supplement: Multimedia component 1 [file mmc1.docx]

Supplemental Table 1. Nutrient contents milk (NHANES food code 11100000; milk, nfs)

| Nutrients | Amount per cup |
| --- | --- |
| Energy | 122 kcal |
| Carbohydrate | 11.7 g |
| Dietary fiber | 0.00 g |
| Total sugars | 12.5 g |
| Added sugars | 0.00 tsp eq |
| Protein | 8.03 g |
| Total fat | 4.83 g |
| Total monounsaturated fatty acid | 1.28 g |
| Total polyunsaturated fatty acid | 0.23 g |
| Total saturated fatty acid | 2.92 g |
| Calcium | 290 mg |
| Magnesium | 27.1 mg |
| Potassium | 358 mg |
| Sodium | 103 mg |
| Vitamin A, RE | 124 µg |
| Folate, DFE | 11.5 µg |
| Vitamin B_12_ | 1.14 µg |
| Vitamin D | 2.99 µg |
